# Covid-19 crisis in the Netherlands: “Only together we can control Corona”

**DOI:** 10.1007/s11299-020-00257-x

**Published:** 2020-09-14

**Authors:** Gerrit Antonides, Eveline van Leeuwen

**Affiliations:** Department of Social Sciences, Wageningen University, Hollandseweg 1, 6706 KN Wageningen, The Netherlands

**Keywords:** Covid-19, Lockdown measures, Economic measures, Consumer behavior

## Abstract

The development and management of the Covid-19 outbreak in the Netherlands is described. The “intelligent lockdown” was aimed at minimizing new infections and limiting the number of deaths, while keeping the economy running as much as possible. Changes in consumer behavior, exit strategy, and lessons learned are considered.

The Dutch government does not want to tell the 17 million population that “they have to do this or that, and it does not want to play the boss” (Dutch Prime Minister 2020). Also, the Dutch government listens to scientists. That is why the Dutch management of the Covid-19 outbreak differs from many other countries. In this contribution, we give an overview of the main events, policies and behaviors associated with the crisis. We also point out some lessons learned and state some expectations for the future.

## Main events

Due to visits abroad and the Dutch carnival in the south of the country, the first Covid-19 infection occurred February 27, 2020, followed by more cases after travelers returned from winter sport in Italy and Austria. The first Covid-19-death was observed March 6. Initially, the Municipal Health Services traced the course of infections, but they had to give it up soon. Massive testing was impossible due to the limited number of available tests, which were reserved for hospital personnel only. Initially, the government seemed to be aiming for group immunity, but later realized this was not feasible.

The first national government measure was a social distancing policy accompanied by sanitary advice and a request to stay at home as much as possible, announced at a press conference on March 9. “Intelligent lockdown measures” were announced March 12, including cancellation of events with over 100 people, limited visits to the elderly and other vulnerable people, and advice to work at home whenever possible. The aim was protecting people against the virus while keeping the economy running as much as possible. After this announcement, churches canceled their services, universities changed to online teaching, many shops temporarily closed, and public transportation was heavily reduced. At regular press conferences (some of which were watched by over 7 million people), further measures were announced, including closing all nursery homes to visitors, limiting the number of funeral visitors to 30, and prohibiting parties of more than 2 people (except household members). Several recreation areas and beach roads were closed to prevent people from gathering too closely, especially after much crowding on that sunny weekend in March. Only necessary businesses, (all) shops and public transportation were allowed to continue their operations if they took into account the health and 1.5 m-distance regulations. Restaurants and bars had to close but were allowed to offer take-away/delivery service of their food products. Although people did not like the measures, the lively sign language interpreter at the press conferences was very popular. On March 20, the king held a speech to support the Dutch people in fighting the disease. Meanwhile, the virus raged on, leading to a peak of 1322 patients at intensive care units on April 7. By the beginning of July, over 6100 confirmed Covid-19-deaths had occurred (the real number being higher because not all deaths were tested). In the beginning of May, an estimated 5.4% of the population had antibodies in their blood (8–10% in the south-east of the Netherlands), far from the amount necessary for group immunity. From June on, everyone having Covid-19 infection symptoms was tested, followed by tracking the source of infection in case of a positive result. In addition to hospital workers also care givers in nursing homes and home care workers were given medical face masks for protection. Early July, the reproduction factor of the virus (R, the number of people infected by one contagious person) was 0.9, the daily number of infected people was approaching zero, and almost no intensive care admissions occurred anymore.

Next to healthcare, the government also invested in the mitigation of the economic consequences of the lockdown. On March 31, an emergency measure to maintain jobs was published, compensating 90% of wages for companies facing low turnover for 3 months (if all personnel remained on the job); the measure was prolonged in June for another 3 months (although this time it was allowed to lay off workers). Also, self-employed people could receive social benefit allowances for 3 months. Furthermore, an extra subsidy of 4000 euros was provided to companies hit by the lockdown measures. Finally, start-up companies could borrow up to 2 million euros to overcome the crisis. Flexible contract workers were given an allowance of 600 euros for 3 months. End of June, airliner KLM was given a one-billion euro loan and state guarantees for another 2.4 billion euro bank loans.

Households were asked to not cancel their subscriptions to sport schools and gyms. Also, childcare, even though not provided, had to be paid for to avoid massive job losses. The government promised to compensate all childcare costs paid by households for the period March–June 8.

Nevertheless, unemployment went up from 2.9% in February to 3.6% in May, an unpreceded increase in such a short time. In total, the Dutch government is expected to borrow an extra 93 billion (11.8% of the 2020 budget) to cover the budget deficit in 2020. The Dutch economy suffered dramatically with so many shops, and hospitality and catering industry closed. The Dutch economy is expected to shrink by 6% in 2020 (CPB 2020). The Dutch stock market index AEX had dropped 35% on March 18 as compared with a month before. Consumer confidence had dropped from − 2 in March to a low of − 31 in May (− 27 in June) (Statistics Netherlands 2020a); producer confidence had dropped from 0.2 in March to − 28.7 in April, the largest drop ever (recovering to − 15.1 in June) (Statistics Netherlands 2020b).

Due to the lockdown measures, many large-scale events were canceled, including football matches, Marathon running events, 4 days marches in Nijmegen, and other sport events, King’s day, 75th anniversary of Liberation Day of WWII, Gay Pride, and music festivals Pinkpop, Lowlands, and North Sea Jazz. With schools and universities closed and festivals canceled, many young people suffered from boredom and impatience to get back to normal, with consequent problems for maintaining the lockdown measures. At several occasions F*** Corona parties and a wedding party with over a hundred guests had to be stopped. Long queues could be observed right before coffeeshops (selling cannabis) had to close down. Fines of 390 euros were given to people who gathered in groups over two persons, and drones were deployed to help maintain social distancing.

## Changes in consumer behavior and daily life

Clearly, the lockdown severely changed the Dutch way of living. Since everyone was supposed to stay at home as much as possible, traffic decreased significantly. The use of public transport decreased to almost zero, and also car usage decreased by half (in terms of distance) in the first weeks of the lockdown. At the same time, people went more often for a walk or bike trip (both doubled in terms of distance).

A survey held by one of the authors that focused on the effect of the Dutch lockdown showed that people in the most urban areas saw a steeper decrease in their well-being and faced more health problems than those in rural areas. At the same time, they also faced a sharp decrease in their level of physical exercise, while this actually increased for many people in the most rural areas.

As often, the most vulnerable people were hit most by the lockdown. And while many feared the impact on lonely elderly, it appeared that also the younger generation was hit severely. In particular students and (young) singles really missed social interaction at school, work, or during leisure time.

Our way of consumption changed as well. Expenditures at the supermarkets went up significantly. To compensate for not being able to visit restaurants and bars, people bought higher quality and more luxury food, such as fresh vegetables and special beers. People also spent more time on preparing their food as well as on baking cakes and cookies. Overall, around 20% of the people indicated they were eating healthier in this period. Also, the demand for local foods increased, resulting in several local and regional initiatives.

At the same time, expenditures on non-food went down for most products. In particular expenditures on cars, clothing, shoes and accessories went down, to a lesser extent furniture and electric appliances, while expenditures on music, books and games, as well as do-it-yourself articles went up. Not surprisingly, online sales went up as well; according to some by as much as 50%. Not only big webshops benefited from the crisis, but also small and local shops went online and benefited. Digitalization developed enormously: restaurants went online, sport schools and physiotherapists offered part of their services online, as did churches, schools and universities. In addition, people reported to use online communication programs to have joint dinners, pub quizzes and beer tasting events, as well as to make music and, of course, to have work meetings.

## Exit strategy

When the number of IC patients dropped below 700, some measures were relaxed. May 11, it was announced that primary schools were to (partly) open again, sports allowed for youth under 12 years of age, swimming pools, contact professions, libraries and fun parks could open, group gatherings under 10 persons were allowed, all taking account of the 1.5 meters physical distance. Experiments allowing visitors at nursery homes were started.

June 1, pubs (guests seated at tables for four), and restaurants opened up (for no more than 30 people, with reservation), secondary schools, museums and theaters were opened, allowing a maximum of 30 visitors. Necessary public transportation was allowed when wearing a face mask, although the government had a hard time explaining why face masks were useful where the 1.5 meters distance norm could not be maintained.

July 1, the maximum number of visitors of facilities inside was increased to 100. Holiday parks and campings, casinos and coffeeshops were opened, and all sports were allowed for everyone, still taking account of 1.5 meters physical distance. Children up to 12 years old were no longer obliged to take 1.5 meters distance (pupils up to 18 years old still would keep distance to adults but not among themselves). Traveling to most EU countries was made possible again, although not for many countries outside the EU.

From September 1, events with more than 100 people might be allowed. Universities will be allowed to provide face-to-face teaching again when complying with the 1.5 m distance rule. Large-scale events should wait until next year.

## Lessons learned

The Covid-19 crisis has taught a number of lessons for the future. Most importantly, readiness to face the pandemic and anticipation of measures needed were minimal, partly due to inertia in government decision making. The initial strategy of aiming for group immunity has been abandoned quickly. This strategy would have caused too many deaths and was little understood by the population. Since the pandemic is abating plans are made to increase the number of intensive care units, and stocks of protecting and testing materials, permanently. Also, preparedness for a second outbreak of the disease seems to be in order. A triage protocol has been developed which, if intensive care capacity becomes severely limited, gives priority to patients with expected short duration of care, followed by priority for health care professionals, and people of younger age.

During the lockdown the effort of medical personnel was highly praised, as expressed in clapping sessions and support banners of people in many places. After years of wage restraint, the general opinion is that hospital personnel deserve a wage raise finally, although so far only a one-time compensation of 1000 euro was offered.

The Covid-19 crisis has revealed great tension between the medical objective of preventing deaths and limiting the societal costs caused by the lockdown measures. The Dutch solution has been the intelligent lockdown, which was aimed at protecting vulnerable people (mostly elderly) while avoiding layoffs, business failures and bankruptcies as much as possible. Although still costly, the Dutch government debt of just below 50% of GDP was a luxury starting point for providing the necessary economic aid to businesses and employees as described above. The Dutch frugality seems to have paid off in the end.

The intelligent lockdown also respected individual responsibility of the citizens, rather than enforcing quarantine and strictly monitoring behavior outside their homes. Similar respect was paid to individual privacy in monitoring the rate of infection in the population. Attempts at developing a mobile app to keep track of the disease all failed because privacy could not be guaranteed (by electronic storage of individual data). Another privacy concern became evident when protests were raised against employers’ attempts to measure temperature or taking medical tests of employees.

The Dutch government gave much economic aid to employees, companies and self-employed people, which has raised some concerns over the economic value of these measures. Apart from its short-term usefulness, the long-term effects may be incentivizing the lack of precautionary behavior of these parties. Examples of such behavior are lack of saving for rainy days, paying exorbitant bonuses to management, paying dividend to stockholders, and lease contracts instead of ownership of production means. Government aid in many such cases may induce moral hazards of more such behavior and expectations of the government bailing out companies in trouble. In a wider perspective, the Dutch government faces similar dilemmas in contributing to unconditional foreign aid to southern European countries.

Although the Netherlands is now more prepared for a pandemic, a second Covid-19 outbreak is still feared. Moreover, hygiene and distancing discipline is fading, especially among the youth. Despite much effort to develop cures and vaccines, they are not expected to become available before 2021. Also, some doubt has been raised with regard to their long-run effectiveness, since antibodies have been shown to disappear within a couple of months.

Finally, despite economic hard times being expected to come, several positive side-effects of the Covid-19 crisis may be expected. People may continue working at home more than before the crisis, with consequent reduction of mobility needs and energy demand. Big companies already report on plans to reduce the use of large office buildings and to open up more, smaller, regional offices. There also seems to be a trend of more interest of certain groups of households in moving to the countryside where houses are larger and more green space is provided. Also, government financial aid to businesses may be accompanied by requirements of green investments, hopefully reducing climate change.

## Conclusion

The Dutch government has taken “intelligent lockdown measures” aimed at protecting vulnerable people against the Covid-19 virus while mitigating economic consequences by compensating wages for companies facing low turnover and providing social benefits for self-employed people. The extra costs, estimated at about 12% of GDP, could be afforded rather easily because of the low government debt of just below 50% of GDP. Although the virus has caused over 6100 deaths, the reproduction factor has been brought down to 0.9, eventually leading to no further infections. Several lessons on how to deal with future outbreaks have been learned.
